# Different Effects of Metformin and A769662 on Sodium Iodate-Induced Cytotoxicity in Retinal Pigment Epithelial Cells: Distinct Actions on Mitochondrial Fission and Respiration

**DOI:** 10.3390/antiox9111057

**Published:** 2020-10-28

**Authors:** Chi-Ming Chan, Ponarulselvam Sekar, Duen-Yi Huang, Shu-Hao Hsu, Wan-Wan Lin

**Affiliations:** 1 Department of Pharmacology, College of Medicine, National Taiwan University, Taipei 100233, Taiwan; arul9600354038@gmail.com (P.S.); tyh123kimo@yahoo.com.tw (D.-Y.H.); 2 Department of Ophthalmology, Cardinal Tien Hospital, New Taipei City 23148, Taiwan; nwokcsky@gmail.com; 3 School of Medicine, Fu Jen Catholic University, New Taipei City 242062, Taiwan; 4 Graduate Institute of Medical Sciences, Taipei Medical University, Taipei 110301, Taiwan; 5 Department and Graduate Institute of Pharmacology, National Defense Medical Center, Taipei 11490, Taiwan

**Keywords:** sodium iodate (NaIO_3_), AMP-activated protein kinase (AMPK), mitochondrial fission, mitochondrial respiration

## Abstract

Oxidative stress-associated retinal pigment epithelium (RPE) cell death is critically implicated in the pathogenesis of visual dysfunction and blindness of retinal degenerative diseases. Sodium iodate (NaIO_3_) is an oxidative retinotoxin and causes RPE damage. Previously, we found that NaIO_3_ can induce human ARPE-19 cell death via inducing mitochondrial fission and mitochondrial dysfunction. Although metformin has been demonstrated to benefit several diseases possibly via AMP-activated protein kinase (AMPK) activation, it remains unknown how AMPK affects retinopathy in NaIO_3_ model. Therefore, in this study, we compared the effects of metformin and AMPK activator A769662 on NaIO_3_-induced cellular stress and toxicity. We found that A769662 can protect cells against NaIO_3_-induced cytotoxicity, while metformin exerts an enhancement in cell death. The mitochondrial reactive oxygen species (ROS) production as well as mitochondrial membrane potential loss induced by NaIO_3_ were not altered by both agents. In addition, NaIO_3_-induced cytosolic ROS production, possibly from nicotinamide adenine dinucleotide phosphate (NADPH) oxidase activation and counteracting cell death, was not altered by A769662 and metformin. Notably, NaIO_3_-induced mitochondrial fission and inhibition of mitochondrial respiration for ATP turnover were reversed by A769662 but not by metformin. In agreement with the changes on mitochondrial morphology, the ERK-Akt signal axis dependent Drp-1 phosphorylation at S616 (an index of mitochondrial fission) under NaIO_3_ treatment was blocked by A769662, but not by metformin. In summary, NaIO_3_-induced cell death in ARPE cells primarily comes from mitochondrial dysfunction due to dramatic fission and inhibition of mitochondrial respiration. AMPK activation can exert a protection by restoring mitochondrial respiration and inhibition of ERK/Akt/Drp-1 phosphorylation, leading to a reduction in mitochondrial fission. However, inhibition of respiratory complex I by metformin might deteriorate mitochondrial dysfunction and cell death under NaIO_3_ stress.

## 1. Introduction

Age-related macular degeneration (AMD) is the leading cause of irreversible blindness in aged peoples in the world. AMD is generally characterized by a permanent loss of retinal pigmented epithelium (RPE), which plays essential functional roles in maintaining retinal homeostasis and supporting the health of photoreceptors [1]. Although the pathophysiology of AMD is still not clearly understood, oxidative stress injury of the RPE is one of the risk factors associated with disease progression [2].

NaIO_3_-induced oxidative stress and subsequent cytotoxicity in RPE cells represent a valuable model to dissect the pathogenesis and therapeutic strategies of AMD. NaIO_3_ is an oxidative toxic agent and its retinal toxicity has been widely studied in cultured RPE cells [3,4,5] and animals in order to evaluate and develop protective agents against RPE cell injury [6,7,8]. In our previous study, we demonstrated that NaIO_3_-induced cell death in RPE cells results from the dramatic mitochondrial fission accompanied by the inhibition of mitochondrial ATP production. We also showed that cytosolic reactive oxygen species (ROS) level is indispensable for autophagy and is involved to protect cells against NaIO_3_ [9].

AMP-activated protein kinase (AMPK), an evolutionarily conserved enzyme, is a master kinase that plays a crucial role in cell responses in many aspects, including metabolism, energy homeostasis, cell growth, inflammation, infection, redox regulation, tissue repair, regeneration etc. [10,11,12]. Likewise, AMPK activity has been demonstrated to protect RPE cells from UV radiation [13], hydrogen peroxide [14], photoreceptor outer segment [15] and hypoxia [16], and in turn delay inherited retinal degeneration [17]. Protection is associated with suppression of oxidative stress [14,18,19], decrease of DNA damage, induction of autophagy [15] and mitochondrial energy production [19], regulation of lipid metabolism [20], as well as activation of Nrf2/HO-1 and Akt pathways [21,22]. Despite accumulating lines of evidence which support the beneficial effect of AMPK activation, so far there is no report directly determining the impact of AMPK activity on NaIO_3_-induced cellular stress. The only highly related study comes from glycyrrhizin, which can protect RPE cells against NaIO_3_-induced damage through activation of Nrf2/HO-1 pathway [22]. Because glycyrrhizin can activate AMPK in other cell types [23,24] and AMPK can mediate Nrf2 signaling [24], the role of AMPK in protection of RPE cells against NaIO_3_ by glycyrrhizin is expected. Besides this, resveratrol protects RPE cells against NaIO_3_ injury via peroxisome proliferator-activated receptor alpha (PPARα) activation and PPARδ conformational change, while the role of AMPK in PPAR activation remains elusive [25]. Most recently antioxidant kaempferol was shown to protect NaIO_3_-induced pathological changes of retinal tissue and retinal cells apoptosis [26]. Similarly, whether AMPK is involved in regulation of ROS status in NaIO_3_ stressed RPE cells has not been explored yet.

Developing therapeutic strategies on the protection of RPE cells from NaIO_3_-induced cytotoxicity remains an unmet need. Metformin is the first-line medication for type 2 diabetes mellitus and is one of the most frequently used drugs. Recently, the beneficial effects of metformin, beyond insulin sensitizing and blood glucose lowering effects, have had impacts on normal tissue protection, cell regeneration and tumor sensitization [27]. The action mechanisms of metformin include activation of AMPK via suppression of mitochondrial complex I [12], inhibition of mitogen-activated protein kinase (MAPK) and Smads [28], as well as induction of antioxidant activity [29]. Owing to its pleiotropic action mechanisms, metformin has become a potential candidate drug in anticancer [30], cardio-protection [31], hepatoprotection [28], anti-inflammation and anti-ageing [32,33]. In this study, we explored the effects of metformin on NaIO_3_-induced cytotoxicity in RPE cells, and elucidated the molecular mechanisms underlying this outcome. Meanwhile we chose the selective AMPK activator A769662 as a comparison to clarify the role of AMPK in NaIO_3_-induced cytotoxicity of RPE cells.

## 2. Materials and Methods

### 2.1. Reagents

NaIO_3_ (sodium iodate), N-acetyl cysteine (NAC), trolox, 3-AB (3-aminobenzamide), DPQ (3,4-dihydro-5-[4-(1-piperidinyl) butoxy]-1(2H)-isoquinolinone), DCFDA (dichlorofluorescein diacetate), DHE (dihydroethidium), oligomycin, FCCP (carbonyl cyanide-p-trifluoromethoxyphenylhydrazone), rotenone, antimycin A, and U0126 were obtained from Sigma-Aldrich Co (St Louis, MO, USA). MitoSOX and Mitotracker green were purchased from Thermofischer scientific (Waltham, MA, USA). MitoPY1 and A769662 were obtained from Tocris Biosciences (Bristol, UK). Metformin was purchased from Medchem express (Monmouth Junction, NJ, USA). The antibodies specific for phospho-ERK1/2 (T202/Y204), ERK1/2, phosphor-Akt (Ser 473), Akt, phospho-dynamin-related protein (DRP)-1 (S616), DRP-1, γH2AX and Tom 20 were purchased from Cell Signaling Technology (Beverly, MA, USA). The β-actin antibody was purchased from Santa Cruz Biotechnology (Santa Cruz, CA, USA). Dulbecco’s Modified Eagle’s Medium/Nutrient Mixture F-12 (DMEM/F12), penicillin, streptomycin and trypsin-EDTA were from Invitrogen (Rockville, MD, USA). The ECL reagent (Western blotting lightening chemiluminescence reagent plus) was purchased from PerkinElmer (Wellesley, MA, USA).

### 2.2. Cell Culture

Human ARPE-19 cells purchased from Food Industry Research and Development Institute (Hsinchu, Taiwan) were maintained in DMEM/F-12 supplemented with 10% fetal bovine serum (FBS), 3.7 g/L NaHCO_3_, 100 U/mL penicillin and 100 μg/mL streptomycin. The cells were cultured in a humidified 5% CO_2_ incubator at 37 °C. For most of the experiments, cells reaching 90–95% of confluence were starved and synchronized in serum-free DMEM/F-12 for 24 h before experiments.

### 2.3. Annexin V-FITC/PI Assay

Cell death assay was measured using Annexin V-FITC Apoptosis Detection Kit (Biolegend, San Diego, CA, USA). ARPE-19 cells were seeded (1 × 10^7^ cells/well) and incubated overnight at 37 °C followed by the indicated treatment. Then cells were suspended in an Annexin V binding buffer and stained together with Annexin V-FITC and propidium iodide (PI) at room temperature for 30 min at 37 °C. Cell samples were placed on ice, away from light, and FITC and PI fluorescence were immediately measured by flow cytometer (BD FACSCalibur, Franklin Lakes, NJ, USA). Percentage of the cells in the respective quadrants was calculated and analyzed by using CellQuest PRO software version 5.1 (BD, Franklin Lakes, NJ, USA).

### 2.4. Determination of Cytosolic H_2_O_2_, Cytosolic Superoxide Anion, Mitochondrial H_2_O_2_ and Mitochondrial Superoxide

Cytosolic H_2_O_2_ and cytosolic superoxide anion (O_2_^−^) were detected using DCFDA and DHE, respectively. Mitochondrial O_2_^−^ and H_2_O_2_ were detected using mitoSOX Red and mitoPY1, respectively. After major stimulation, ARPE-19 cells were washed with phosphate-buffered saline (PBS) and incubated with 10 μM DCFDA, 5 μM DHE, 5 μM MitoSOX Red or 5 μM mitoPY1 at 37 °C for 30 min. Subsequently, the cells were washed in PBS, trypsinized and the fluorescence intensity was measured by flow cytometry (FACS calibur, Franklin Lakes, NJ, USA) at excitation/emission wavelengths of 485/530 nm, 510/595 nm, 510/580 nm and 503/528 nm for DCFDA, DHE, mitoSOX and mitoPY1, respectively. For each sample, ROS production was expressed as a percentage of control from the same experiment.

### 2.5. Western Blot Analysis

After major treatment, whole cell lysate extracts were harvested, sonicated and centrifuged, and equal protein amounts as determined by the Bradford protein assay were subjected to SDS-PAGE and transferred to a polyvinylidene difluoride membrane. After incubation with 5% nonfat milk in tris-buffered saline (TBST) (10 mM Tris, pH 8.0, 150 mM NaCl, 0.5% Tween 20) for 60 min, the membrane was washed once with TBST and incubated with specific antibodies at 4 °C for 12 h. Membranes were washed three times with TBST for 10 min and incubated with a horseradish peroxidase-conjugated anti-mouse or anti-rabbit antibodies (1:5000 dilution) for 2 h. Blots were then washed with TBST three times and detected with enhanced chemiluminescence detection reagent. Equal amounts of sample protein loading were standardized by using β-actin as the internal control.

### 2.6. Measurement of Mitochondrial Oxygen Consumption Rate

The oxygen consumption rate (OCR) was measured by the extracellular flux analyzer XF24 (Agilent technologies, CA, USA) as we previously described [9]. Cells (4 × 10^5^ cells/well) were plated in Seahorse 24-well V7 microplates and cultured in complete DMEM/F-12 medium for 24 h in a 5% CO_2_ incubator at 37 °C. Then, the cells were incubated in an XF assay medium without NaHCO_3_ and FBS for 1 h at 37 °C in a measuring chamber without CO_2_ input. The mitochondrial complex inhibitors oligomycin (2.5 μΜ), FCCP (1 μM), antimycin A (2.5 μM)/rotenone (2.5 μM) were individually injected at 26 min, 50 min and 74 min after a starting measurement of the basal respiration, respectively. OCR was recorded as pMoles per minute. Averages of three wells were taken per data point.

### 2.7. Mitochondrial Imaging

Mitochondrial imaging was measured as we previously described [9]. After drug treatment, cells were fixed with 4% paraformaldehyde at 37 °C followed by permeabilization with 0.2% Triton X-100 for 15 min and blocking by 5% bovine serum albumin (BSA) and normal IgG (1:300) for 1 h. For mitochondrial morphology, cells were treated with Tom20 antibody (Abcam, Cambridge, UK) in 1% BSA overnight at 4 °C. After washing with PBS, cells were incubated with secondary antibody in 1% BSA in PBS for 1 h at room temperature and then mounted with DAPI Fluoromount-G (Southern Biotech, Birmingham, AL, USA). Images were acquired using a 100 X Plan-Neofluar oil objective of LSM 880 in Airyscan SR microscopy (Carl Zeiss Micro Imaging GmbH, Jena, Germany).

### 2.8. Statistical Analysis

All data presented as mean ± standard error mean (S.E.M.) were obtained from at least three independent experiments. Multiple groups were compared by one-way analysis of variance and Bonferroni post-test, making use of Graph pad software (Graph Pad Software, San Diego, CA, USA). Two groups were compared with an unpaired Student’s *t* test and two-tail *p* value. Results were considered statistically significant when *p* < 0.05.

## 3. Results

### 3.1. Cytosolic ROS Production Counteracts Cell Death under NaIO_3_ Treatment

**Figure 1:**
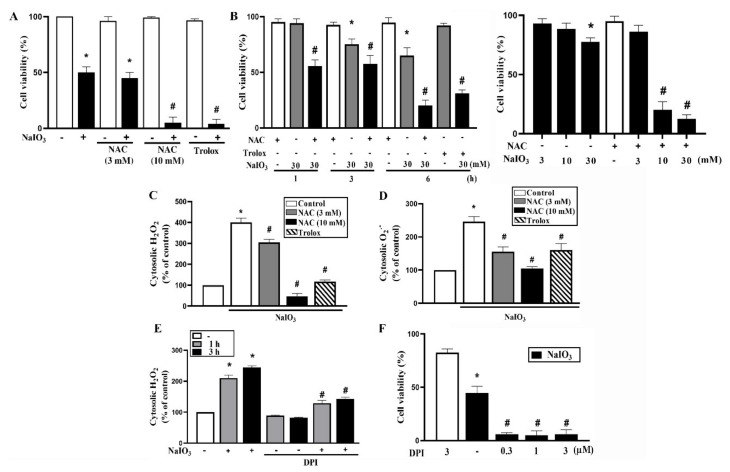
NaIO_3_-induced cell death is enhanced by antioxidants N-acetyl cysteine (NAC) and trolox. (**A**) Cells were pretreated with NAC (3 mM, 10 mM) 20 min prior to NaIO_3_ (30 mM) for 18 h. (**B**) Cells were pretreated with NAC (10 mM), trolox (500 μM) 20 min prior to NaIO_3_ stimulation for indicated time points (left panel) or 6 h (right panel). Cell viability was determined by Annexin V/PI staining using flow cytometry. (**C**,**D**) After pre-treatment with antioxidants followed by NaIO_3_ (30 mM) for 6 h, cytosolic H_2_O_2_ (**C**) and cytosolic O_2_^−^ (**D**) were measured by using DCFDA and DHE, respectively. (**E**) After pretreatment with DPI (3 μM) 20 min prior to NaIO_3_ (30 mM) for indicated time points, cytosolic H_2_O_2_ was determined. (**F**) Cell viability was determined after cells were pretreated with DPI followed by NaIO_3_ (30 mM) for 18 h. Data were mean ± SEM from three independent experiments. * *p* < 0.05, indicating the significant effect of NaIO_3_. ^#^ *p* < 0.05, indicating the significant enhancement effects of NAC, trolox and DPI on the response of NaIO_3_.

Cytosolic ROS mediates diverse cellular processes to control cell viability. Our and other studies have shown that NaIO_3_ can induce cytosolic ROS production and lead to oxidative stress in RPE cells [9,18,25]. Here we first examined the effects of antioxidants NAC and trolox on the responses of NaIO_3_. We unexpectedly found that both antioxidants can exacerbate cell death after NaIO_3_ treatment for 18 h. Interestingly, this exacerbation was only observed of NAC at 10 mM but not at 3 mM (Figure 1A). In order to understand how fast cell death was enhanced under antioxidant treatment, we incubated cells for short period. Our data revealed that in the presence of NAC (10 mM) or trolox (500 μM), NaIO_3_ (30 mM) can dramatically induce cell death up to 80% after 6 h incubation (Figure 1B, left panel). Accordingly, NAC (10 mM) can sensitize cells to a sub-toxic concentration of NaIO_3_ (10 mM) at 6 h (Figure 1B, right panel). These findings suggest that optimal cytosolic ROS production confers a protection against NaIO_3_ stress.

To verify this notion, we determined cytosolic ROS level by using DCFDA and DHE, which primarily measure H_2_O_2_ and O_2_^−^, respectively. When using DCFDA, we found that NAC (3 and 10 mM) can reduce cytosolic ROS at resting state, but only higher concentration of NAC (10 mM) is sufficient to abolish NaIO_3_-induced cytosolic ROS production (Figure 1C). This finding might explain why NAC (3 mM) cannot protect cells against NaIO_3_-induced cell death possibly being due to the weak inhibition on cytosolic ROS production. Similarly, trolox (500 μM) can dramatically reduce basal and NaIO_3_-induced cytosolic ROS production (Figure 1C). When using DHE, we observed similar effects of NAC and trolox to reduce cytosolic ROS production caused by NaIO_3_ as observed when using DCFDA (Figure 1D). These findings support our notion that cytosolic ROS is required for protecting cells upon NaIO_3_ stress.

To understand whether nicotinamide adenine dinucleotide phosphate (NADPH) oxidase is the origin of NaIO_3_-induced cytosolic ROS, we examined the effects of DPI (an inhibitor of cytosolic NADPH oxidase) together with A769662. As the effects of NAC and trolox, DPI (3 μM) can reduce NaIO_3_-induced cytosolic ROS production (Figure 1E) and concomitantly enhance the cell death at 6 h and 18 h (Figure 1F). Therefore, these findings further strengthen the crucial role of cytosolic ROS to protect RPE cells against NaIO_3_ stress. Moreover, NADPH oxidase is the major cytosolic ROS source of NaIO_3_.

### 3.2. PARP1 Is Not Involved in NaIO_3_-Induced Cell Death

Considering that ROS production might induce DNA damage and contribute to cell viability, we assessed the role of PARP1 in NaIO_3_-induced cell death. Nuclear PARP1 activated by DNA damage can modulate cell viability. First, NaIO_3_ was previously found to quickly increaseγ-H2AX formation at 30 min [9]. Notably, we found that NAC can facilitate and prolong NaIO_3_-induced γ-H2AX formation (Figure S1A), suggesting NAC might enhance DNA damage and subsequent PARP1 activation. Nevertheless, PARP1 inhibitors 3AB (50 μM) and DPQ (25 μM) cannot alter cytotoxicity induced by NaIO_3_ (Figure S1B). These findings indicate that although NAC can increase NaIO_3_-induced DNA damage and caspases activation, PARP1 activity is not involved in the cytotoxic action of NaIO_3_.

### 3.3. A769662 Protection While Metformin Enhancement of NaIO_3_-Induced Cytotoxicity Are Unrelated to Cytosolic ROS Production

**Figure 2:**
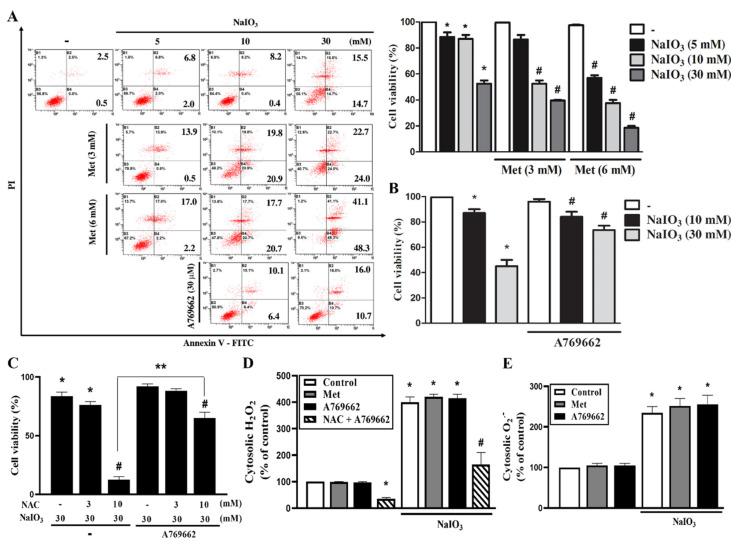
A769662, but not metformin, reverses the NaIO_3_-induced cytotoxicity. Cells were pretreated with metformin (3 mM, 6 mM) (**A**), A769662 (30 μM) (**B**) or NAC (3, 10 mM) (**C**) followed by NaIO_3_ stimulation in different concentrations for 18 h. Cell viability was determined by Annexin V/PI staining using flow cytometry. (**D**,**E**) Cells were pretreated with metformin (6 mM) or A769662 (30 μM) prior to NaIO_3_ stimulation. Cytosolic H_2_O_2_ (**D**) and cytosolic O_2_^−^ (**E**) were measured by using DCFDA and DHE, respectively. Data were mean ± SEM from three independent experiments * *p* < 0.05, indicating the significant effects of NaIO_3_ to induce cell death and ROS production. ^#^ *p* < 0.05, indicating the significant effects of metformin, A769662 and NAC on the response of NaIO_3_. ** *p* < 0.05, indicating the significant combinational effects of NAC and A769662 on the response of NaIO_3_.

In determining the cell viability by Annexin V/PI flow cytometry, our data indicated that after 18 h of treatment, NaIO_3_-induced cell death was enhanced by metformin at 3 or 6 mM, and this enhancement effect of metformin displayed the concentration dependency (Figure 2A). In contrast, A769662 (30 μM) exerted a protective effect against cell death induced by NaIO_3_ (Figure 2B). We wonder if the opposite actions of metformin and A769662 in NaIO_3_ stress are related to cytosolic ROS, so we determined their effects on cytosolic ROS production, either in the absence or the presence of antioxidants. We also wondered if antioxidants might affect the cell protective action of A769662. Notably, the cell death enhancement caused by NAC (10 mM) was antagonized by A769662 (Figure 2C), while NAC (3 mM) still cannot change cell viability in the absence or the presence of A769662 (Figure 2C). Moreover, we found that the NaIO_3_-induced cytosolic ROS increase at 3 h was not changed by A769662 (30 μM) or metformin (6 mM) by either DCFDA (Figure 2D) or DHE staining (Figure 2E). In addition, under A769662 treatment, the NaIO_3_-induced cytosolic ROS level still can be inhibited by NAC (10 mM) (Figure 2D). These findings suggest that the cytoprotective action of A769662 and the death promoting action of metformin are unrelated to the changes of cytosolic ROS.

### 3.4. A769662 and Metformin do not Affect Mitochondrial ROS Production nor Mitochondrial Membrane Potential Under NaIO_3_ Stress

Because mitochondrial ROS increase can impair and decrease the mitochondrial membrane potential (MMP), we further addressed the effects of A769662 and metformin on mitochondrial ROS and MMP. Using mitoSOX staining, we found that NaIO_3_ did not alter the mitochondrial O_2_^−^ level at 3 h as we previously reported [9], regardless of A769662 being present or not (Figure S2A). Moreover, NAC (10 mM) cannot affect mitochondrial O_2_^−^ level (data not shown). In contrast, the fluorescence data of mitoPY1 revealed that NaIO_3_ can increase mitochondrial H_2_O_2_, and this effect was not reduced by A769662 or metformin (Figure S2B).

Next, we used two different dyes, JC-1 and rhodamine 123, for measuring MMP. Previously JC-1 was reported to be a more selective and reliable fluorescent probe in MMP measurement than rhodamine 123 [34]. As shown in Figure S2C, the data with JC-1 staining revealed that NaIO_3_ (30 mM) can inhibit MMP at 3 and 6 h, and this effect was neither affected by A769662, nor metformin. Similarly, data of rhodamine 123 staining revealed no effects of A769662 and metformin on NaIO_3_-induced MMP loss (Figure S2D). All these findings on mitochondrial ROS and MMP suggest that both A769662 and metformin do not affect mitochondrial H_2_O_2_ production and mitochondrial membrane depolarization caused by NaIO_3_.

### 3.5. A769662 but not Metformin Restores Mitochondrial Respiration in NaIO_3_-Treated RPE Cells

**Figure 3:**
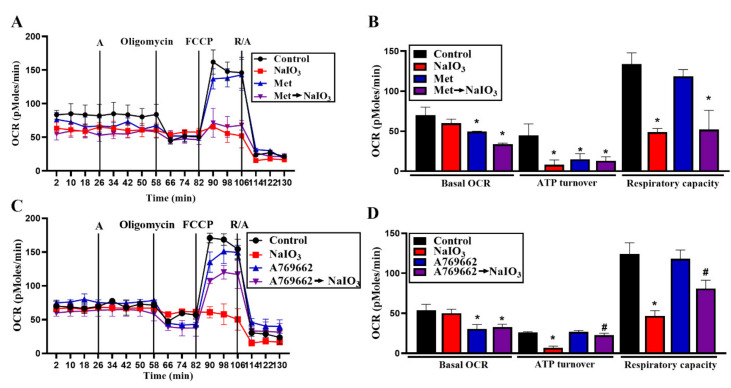
A769662, but not metformin, reverses the NaIO_3_-induced mitochondrial oxygen consumption rate (OCR) decrease. Metformin (6 mM) (**A**) or A769662 (30 μM) (**C**) were pretreated 26 min prior to NaIO_3_ injection through Port A. Then cells were subsequently treated with oligomycin, FCCP and antimycin A/rotenone. Intracellular OCR was measured by seahorse XF24 analyzer. (**B**,**D**) Resting OCR, ATP turnover and respiratory capacity were analyzed and the data were mean ± SEM from three independent experiments. * *p* < 0.05, indicating the significant effects of NaIO_3_. ^#^ *p* < 0.05, indicating the inhibitory effects of A769662 on the action of NaIO_3_.

Because severe mitochondrial fission leads to impairment of mitochondrial respiration in NaIO_3_-treated RPE cells [9], we compared the effects of A769662 and metformin on oxidative status in NaIO_3_-treated cells. We found that short term treatment with NaIO_3_ for 22 min did not affect the resting OCR but significantly reduced ATP turnover and respiratory capacity (Figure 3A–D). Metformin and A769662 themselves alone can slightly inhibit resting OCR and respiratory capacity (Figure 3B,D); however, only metformin can reduce ATP turnover (Figure 3B,D). In cells pretreated with each agent followed by NaIO_3_, the resting OCR was still inhibited, while inhibition of ATP turnover and respiratory capacity were partially reversed by A769662 but not by metformin (Figure 3B,D). These data suggest that the partial recovery of mitochondrial ATP production and respiratory capacity might contribute to the protective action of A769662 against NaIO_3_.

### 3.6. Mitochondrial Complex I Inhibition Increases NaIO_3_-Induced Cell Death

**Figure 4:**
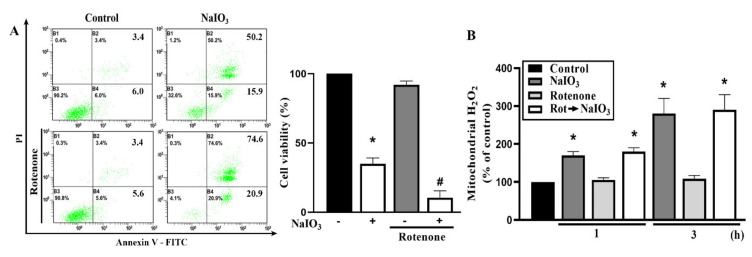
Rotenone enhances the NaIO_3_-induced retinal pigment epithelium (RPE) cytotoxicity. Cells were treated with rotenone (10 μM) 20 min prior to NaIO_3_ (30 mM) stimulation. Cell viability was measured by Annexin V/PI staining (**A**) and mitochondrial H_2_O_2_ was determined by mitoPY1 staining (**B**). Data were mean ± SEM from three independent experiments. * *p* < 0.05, indicating the significant effects of NaIO_3_. ^#^ *p* < 0.05, indicating the significant effect of rotenone to increase cell death under NaIO_3_ stimulation.

Because metformin can inhibit mitochondrial complex I, we wonder if this action might counteract the potential protective action of metformin as A769662 does. Therefore, we determined the effect of rotenone (10 μM) on NaIO_3_ response. Our data revealed a slight but significant enhancement of NaIO_3_-induced cell death in the presence of rotenone (Figure 4A), while mitochondrial ROS induced by NaIO_3_ was not changed by rotenone as we observed for metformin (Figure 4B). The data of rotenone indicate that the inhibition of complex I might contribute to the death enhancement action of metformin.

### 3.7. A769662, but not Metformin, Inhibits NaIO_3_-Induced Mitochondrial Fission via Inhibition of ERK/Akt-Dependent Drp-1 Phosphorylation

**Figure 5:**
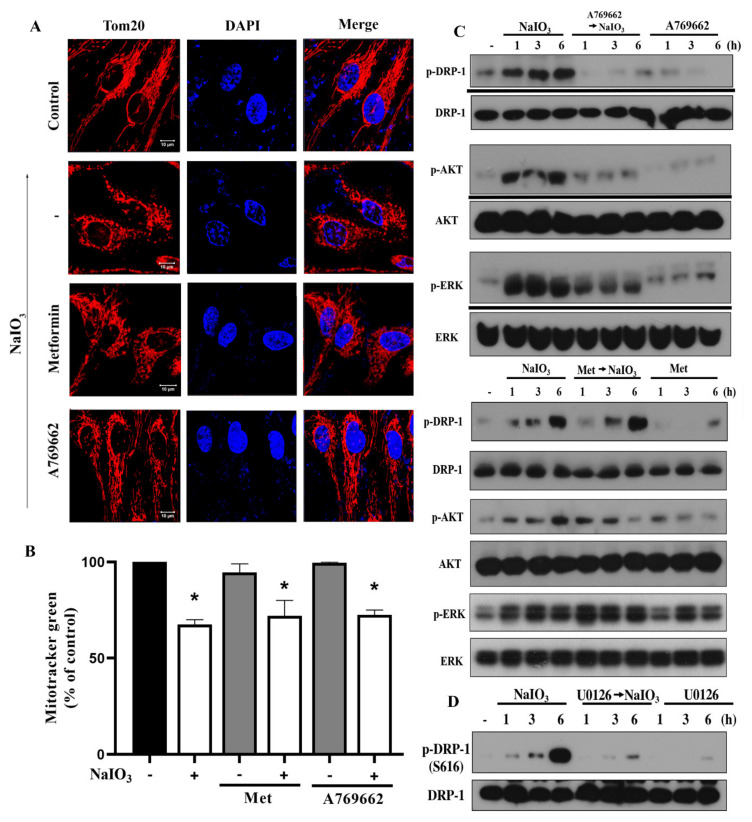
A769662 but not metformin reverses the NaIO_3_-induced mitochondrial fission and Drp-1 phosphorylation. After treatment with metformin (6 mM), A769662 (30 μM) and/or NaIO_3_ (30 mM) for 6 h, Tom20 and DAPI staining were conducted. Scale bars indicated 10 μm (**A**). Mitochondrial mass was determined by Mitotracker green staining followed by flow cytometry (**B**). Data were mean ± SEM from three independent experiments. * *p* <0.05, indicating the significant effect of NaIO_3_. (**C**,**D**) After treatment with indicated agents, cell lysates were prepared for immunoblotting. Data were representative of three independent experiments.

Despite above findings showing no significant effects of A769662 and metformin on NaIO_3_-induced ROS production and mitochondrial membrane potential, we are interested to examine the mitochondrial mass and dynamics. In our previous study we found that NaIO_3_-induced cytotoxicity results from exacerbated mitochondrial fission [9]. Here, using Tom20 as the mitochondrial marker, we found that NaIO_3_-induced mitochondrial fission was completely reversed by A769662, but was not affected by metformin (Figure 5A). Using mitotracker green staining, our data revealed that NaIO_3_ can time-dependently decrease mitochondrial mass, and such inhibition was not altered by metformin or A769662 (Figure 5B).

Moreover, as to mitochondrial dynamic change, we previously found that NaIO_3_ can induce Drp-1 (S616) phosphorylation, an index of mitochondrial fission, but does not affect the major regulators of mitochondrial fusion such as mitofusion 1/2 and OPA1 [9]. As shown in Figure 5C, NaIO_3_-induced Drp-1 phosphorylation was unaffected by metformin but was attenuated by A769662. Next, we elucidated the mechanisms involved to regulate Drp-1 phosphorylation. Our previous study indicated that Akt is involved in NaIO_3_-induced Drp-1 phosphorylation and mitochondrial fission, and ERK mediates Akt activation [9]. In addition, ERK has also been shown to directly phosphorylate Drp-1 at S616 [35,36]. Therefore, we determined the effects of A769662 and metformin on NaIO_3_-induced signal pathways. We found that A769662 but not metformin can inhibit NaIO_3_-induced ERK and Akt phosphorylation (Figure 5C), and MEK inhibitor U0126 can inhibit Drp-1 response of NaIO_3_ (Figure 5D). All these findings suggest that attenuation of ERK/Akt-dependent Drp-1 phosphorylation and mitochondrial fission contribute to the protection effect of A769662.

## 4. Discussion

RPE working as the outer blood retina barrier is vulnerable to oxidative stress. NaIO_3_ is an oxidative retinotoxin and can cause RPE cell damage via inducing aberrant mitochondrial fission and suppressing mitochondrial respiration, leading to mitochondrial dysfunction. Because metformin has been implicated in multifaceted actions and might have nonglycemic benefits, for example in chronic kidney disease [37], lifespan extension [38], anticancer [38], cardiovascular protection and neuroprotection [39], we were interested to address its actions in RPE cells under pathologically oxidative conditions. Moreover, metformin is one of the well identified pharmacological agents to activate AMPK, which possibly is subsequent to its action in inhibition of respiratory complex I [40]. In addition, although AMPK might be a potential therapeutic target for AMD [41], its beneficial action mechanism beyond suppression of oxidative stress in RPE cells remains unclear. Therefore, to understand the AMPK-dependent and -independent actions of metformin, we compared metformin actions in NaIO_3_-treated RPE cells with a selective AMPK activator A769662 [42].

Mitochondrial dynamics play a crucial role in multiple cellular responses. Dysregulation of mitochondrial structural changes, i.e., the balance of mitochondrial fusion and fission, leads to impaired cellular metabolism, ROS production, ionic fluxes and cell viability. Upon facing mitochondrial dynamic stress, mitophagy induction has been implicated to mediate cell protection by clearing damaged mitochondria and maintain the mitochondrial functions and quality for the requirement level [43]. Previously we have demonstrated the involvement of mitochondrial fission in NaIO_3_-induced RPE cell death, and the protective function of mitophagy activated by cytosolic ROS production to ameliorate mitochondrial fission and counteract the death processes [9]. In this study, we further confirm the role of cytosolic ROS in the protection of cells against NaIO_3_ stress. We found that NAC and trolox, both of which decrease cytosolic ROS level, can enhance cell death under NaIO_3_ treatment. Moreover, the NADPH oxidase inhibitor DPI similarly exerts an increase in NaIO_3_-induced cell death. Although AMPK activation has been shown to decrease oxidative stress in RPE cells in response to H_2_O_2_ [14,21], all-trans-retinal [18], bright light [17] and UV [13], we do not observe effects of A769662 and metformin on NaIO_3_-induced ROS production in RPE cells. This means the actions of AMPK might depend on the cellular context caused by various stress inducers, and multifaceted molecular mechanisms are regulated by AMPK.

Previously, oxidative stress has been shown to activate PARP-1 in ARPE-19 cells, contributing to cell necrosis via an AIF-independent parthanatos [44]. In this study, although we for the first time show that the DNA damage event under NaIO_3_ was enhanced by NAC, we do not observe any effects of PARP1 inhibitors (3AB and DPQ) on NaIO_3_-induced cytotoxicity. This finding suggests that DNA damage caused by NaIO_3_ does not contribute to cell death process. Another finding under NaIO_3_ treatment is observing the increase of mitochondrial H_2_O_2_ but not O_2_^−^. Currently we do not have clear evidence to answer such findings, but whether mitochondrial SOD (superoxide dismutase) is involved in this event needs further investigation.

In this study, we unexpectedly observe the differential actions of metformin and A769662 in cell viability under NaIO_3_ treatment. A769662 can exert cell protection against NaIO_3_, even in the higher stress condition of NAC presence. In contrast, metformin exerts a concentration-dependent effect to deteriorate cell viability in response to NaIO_3_. Our ROS data revealed that both opposite actions of metformin and A769662 are not resulting from the changes of cytosolic and mitochondrial ROS, nor the loss of mitochondrial membrane potential. Even in the condition with NAC treatment to attenuate cytosolic ROS, A769662 still exerts cell protection, further suggesting its action independent of ROS. On the other hand, NaIO_3_-induced attenuation of mitochondrial function for ATP turnover and mitochondrial respiratory capacity are reversed by A769662 but not by metformin. We further used rotenone to show its death enhancement effect on NaIO_3_, without changing mitochondrial ROS production. These findings suggest that inhibition of mitochondrial complex I might be involved in the death enhancement action of metformin by decreasing ATP production. On the other hand, A769662 restores mitochondrial respiration ability and capacity for ATP production might contribute to cell protection.

Besides mitochondrial respiration, we also show an alternative action for the distinctive effects of metformin and A769662 in response to NaIO_3_ in RPE cells. We previously observed that increased mitochondrial fission leads to mitochondrial dysfunction in NaIO_3_-treated RPE cells [9]. Drp-1 is a crucial molecule to promote mitochondrial fission, and its activity is regulated by a variety of post-translational modifications. Among them, phosphorylation at S616 is a key modification, and can be achieved by several kinases, including Akt and ERK. Akt has been shown to mediate Drp-1 phosphorylation and mitochondrial fission, leading to neuronal cell death [45,46]. Likewise, ERK-mediated Drp-1 phosphorylation [35,47]. In our previous study, we have demonstrated the ability of NaIO_3_ to induce ERK-Akt signaling axis in RPE cells [9]. In this study, we further extend this signaling pathway to Drp-1 phosphorylation, and show the differential actions of A769662 and metformin in Drp-1. We found that A769662 but not metformin can concomitantly attenuate NaIO_3_-induced ERK and Akt activities as well as Drp-1 phosphorylation and mitochondrial fission. Currently we do not have a clear explanation for this difference in signaling cascade between metformin and A769662, but AMPK activation vs. mitochondrial complex I inhibition might be the potential reason, and it needs further investigation in the future.

AMPK is a heterotrimeric enzyme and a prominent regulator of metabolic homeostasis by monitoring cellular energy status. AMPK activation provides beneficial outcomes in metabolic disorders like insulin resistance-associated type 2 diabetes. In particular, AMPK is involved to control mitochondrial physiology, including mitochondrial biogenesis by regulating gene transcription, mitochondrial dynamics by regulating fusion and fission progresses, and mitochondrial turnover by mitophagy [48]. Therefore, AMPK can mediate adaptive responses to oxidative stress-associated mitochondrial dysfunction [49]. Concomitant activation of AMPK and improved expression of proteins related to mitochondrial dynamics such as MFN-2 and OPA1 have been demonstrated in cells with mitochondrial dysfunction [50] or treatment with resveratrol [51]. The latter study of resveratrol was shown to ameliorate disorders of mitochondrial biogenesis and dynamics via activation of AMPK/PGC-1α signaling pathway in a rat chronic ocular hypertension model [51]. To date, the role of AMPK in the protection of NaIO_3_-induced retinopathy remains limited. The related reports in this aspect include glycyrrhizin, which can protect RPE cells against NaIO_3_-induced retinal injury through activation of Akt and Nrf2/HO-1 pathway [22]. Because glycyrrhizin is an AMPK activator which can transduce Nrf2/HO-1 downstream signal [23], the contribution of AMPK in protection of RPE cells is expected. Our findings indicate that the structural change of mitochondria is consistent with altered cellular metabolism. NaIO_3_-elicited change with higher mitochondrial fission is accompanied by less ATP production, and AMPK activator A769662 can diminish both events. In contrast, metformin has no significant protection in these events. Therefore, we suggest AMPK activity relating to mitochondrial dynamics could be the possible mechanism underlying the A769662′s mitochondrial protection, while inhibition on mitochondrial complex I might again contribute to the effect of metformin. In addition to depleting cellular energy through inhibition of the respiratory chain, metformin also was reported to inhibit NADPH oxidase in an AMPK-independent manner [52]. Therefore, whether other non-specific actions of metformin might contribute to regulate cell viability and signaling in NaIO_3_-treated RPE cells needs further investigation. Taken together, our study suggests that AMPK activation by targeting mitochondrial dynamics, respiration and biogenesis becomes a new therapeutic strategy in AMD. However, applying metformin in oxidative stress conditions of ocular diseases needs to be treated with more caution.

## 5. Conclusions

AMPK activator can exert cell protection in NaIO_3_-treated RPE cells via inhibition of mitochondrial fission, restoration of oxidative phosphorylation and enhancement of mitochondrial biogenesis. Although metformin is an AMPK activator, it cannot protect RPE cells from oxidative stress caused by NaIO_3,_ and inhibition of mitochondrial complex I might contribute to this action of metformin.

## Supplementary Materials

The following are available online at https://www.mdpi.com/2076-3921/9/11/1057/s1, Figure S1: NAC enhances DNA damage in NaIO_3_ treated RPE cells. Figure S2: A769662 and metformin do not affect NaIO_3_-induced mitochondrial H_2_O_2_ production nor decreased mitochondrial membrane potential.
