# Supplementary material for: Different Effects of Metformin and A769662 on Sodium Iodate-Induced Cytotoxicity in Retinal Pigment Epithelial Cells: Distinct Actions on Mitochondrial Fission and Respiration
Source: Antioxidants (Basel). 2020 Oct 28;9(11):1057. doi: 10.3390/antiox9111057 (PMC7693507; doi:10.3390/antiox9111057)
Supplement: Supplementary file 1 [file antioxidants-09-01057-s001.pdf]

Supplementary figures:

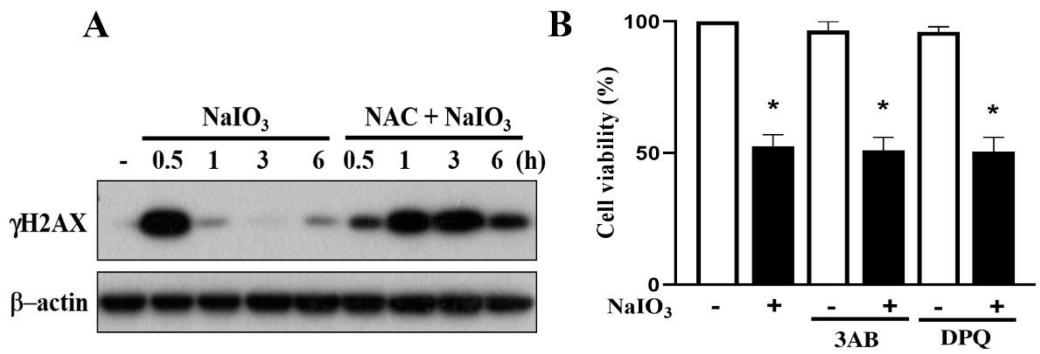

Supplementary Figure S1. NAC enhances DNA damage in $NaIO_3$ treated RPE cells. (**A**) Cells were pretreated with NAC (10 mM) followed by $NaIO_3$ stimulation at indicated time points. γH2AX protein was determined by immunoblotting. (**B**) Cells were pretreated with 3AB (50 μM) or DPQ (25 μM) for 20 min followed by $NaIO_3$ (30 mM) for 18 h. Cell viability was determined by Annexin V/PI staining using flow cytometry. Data were mean ± SEM from three independent experiments $*p < 0.05$, indicating the cytotoxic effects of $NaIO_3$.

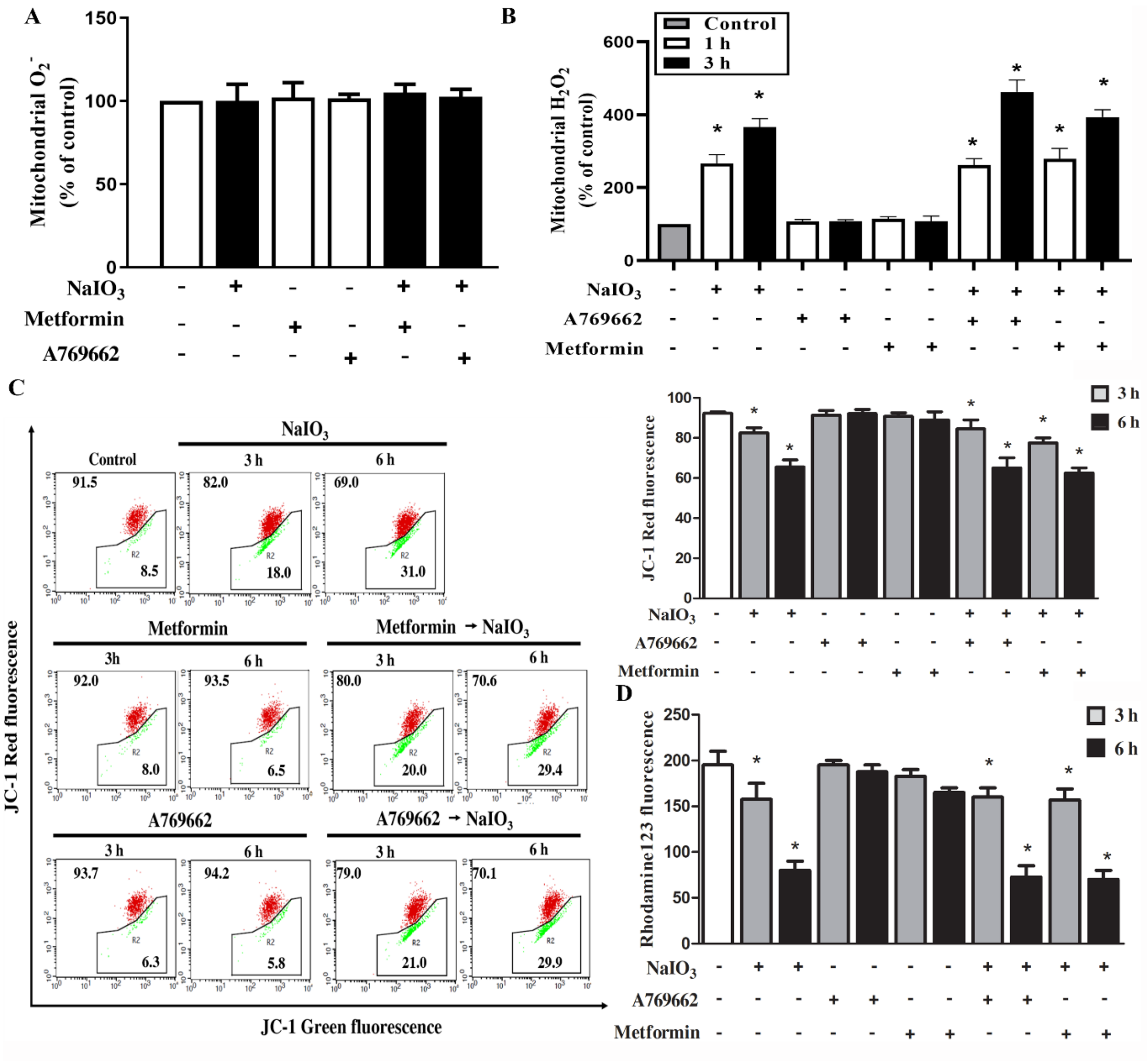

Supplementary Figure S2. A769662 and metformin do not affect $NaIO_3$-induced mitochondrial H_2O_2 production nor decreased mitochondrial membrane potential. Cells were pretreated with metformin (6 mM) or A769662 (30 μM) 20 min prior to $NaIO_3$ (30 mM) stimulation for the indicated times. Mitochondrial O_2^- **(A)** and mitochondrial H_2O_2 **(B)** were determined by mitoSOX and mitoPY1, respectively. Mitochondrial membrane potential was determined by JC-1 **(C)** and rhodamine123 **(D)**, respectively. Data were mean ± SEM from three independent experiments * $p < 0.05$, indicating the significant effects of $NaIO_3$ to induce mitochondrial H_2O_2 production and mitochondrial membrane potential loss.
